# Artificial Intelligence in Stroke Imaging: A Comprehensive Review

**DOI:** 10.5152/eurasianjmed.2023.23347

**Published:** 2023-12-01

**Authors:** Ilker Ozgur Koska, Alper Selver

**Affiliations:** 1 Department of Radiology, Behçet Uz Children’s Hospital, İzmir, Turkey; 2 Department of Advanced Biomedical Technologies, Dokuz Eylül University, İzmir, Turkey; 3 İzmir Health Technologies Development and Accelerator (BioIzmir), Dokuz Eylül University, İzmir, Turkey; 4 Department of Electrical and Electronics Engineering, Dokuz Eylül University, İzmir, Turkey

**Keywords:** Artificial intelligence, convolutional neural network, deep learning, infarct region, radiomics, stroke

## Abstract

The aging population challenges the health-care system with chronic diseases. Cerebrovascular diseases are important components of these chronic conditions. Stroke is the acute cessation of blood in the brain, which can lead to rapid tissue loss. Therefore, fast, accurate, and reliable automatic methods are required to facilitate stroke management. The performance of artificial intelligence (AI) methods is increasing in all domains. Vision tasks, including natural images and medical images, are particularly benefiting from the skills of AI models. The AI methods that can be applied to stroke imaging have a broad range, including classical machine learning tools such as support vector machines, random forests, logistic regression, and linear discriminant analysis, as well as deep learning models, such as convolutional neural networks, recurrent neural networks, autoencoders, and U-Net. Both tools can be applied to various aspects of stroke management, including time-to-event onset determination, stroke confirmation, large vessel occlusion detection, diffusion restriction, perfusion deficit, core and penumbra identification, affected region segmentation, and functional outcome prediction. While building these AI models, maximum care should be exercised in order to reduce bias and build generalizable models. One of the most important prerequisites for building unbiased models is collecting large, diverse, and quality data that reflects the underlying population well and splitting the training and testing parts in a way that both represent a similar distribution. Explainability and trustworthiness are other important properties of machine learning models that could be widely adopted in clinical practices.

**Main Points**

- Stroke is one of the most extensively studied topics in radiology and artificial intelligence (AI) research.
- The urgency of assessing the lesions and the time-critical nature of the disease make stroke attractive for AI research.
- Early stroke detection, large vessel occlusion, diffusion-weighted imaging—Alberta stroke program early computed tomography score estimation, ischemic ore and penumbra estimation, and prognosis prediction are the most important clinical applications of AI in stroke.
- Classical machine learning and deep learning can be applied to stroke; however, with deep learning, more refined outcomes can be obtained.
- Explainability and trustworthiness remain the most important issues to be tackled before large-scale adoption of AI models in stroke.

## Introduction

Stroke is one of the leading causes of death globally. About 6.1% of the total 565 594 deaths in Turkey in 2021 were related to acute stroke.^1^ Every 15 minutes, 1 person dies in Turkey due to stroke.^1^ Furthermore, the cost of stroke to the health-care system is great as well. According to a statistic in the USA, the annual cost of stroke to the health-care system was $56.5 billion between 2018 and 2019.^2^ Due to these facts and the potential benefit of early intervention, there is great interest in the medical image analysis community to develop automatic stroke analysis systems from imaging.^3^ Digital health care is a rising topic among researchers.^4,5^ A total of 214 applications for radiological imaging with artificial intelligence (AI) were approved by the U.S. Food and Drug Administration and/or CE so far. Of these 214 applications, 73 are related to neuroimaging, and more than half of these 73 applications are related to stroke assessment, which is evidence of the great interest of researchers in stroke management automation tasks.^3^ Classical machine learning techniques such as support vector machines,^6^ random forests,^7^ decision trees,^8^ logistic regression,^9^ linear discriminant analysis,^10^ and artificial neural networks^11^ as well as deep learning methods were utilized for these tasks. The exclusion of artifacts from the image is also an important preprocessing step since it may interfere with the quality of quantitative methods.^12^

Classical machine learning methods use hand-crafted features and data science principles, which hierarchically evaluate the feature subsets to find the best subset with respect to the curse of dimensionality.^13^ Curse of dimensionality refers to the importance of dimension of representation vector considering the number of samples in order to prevent overfitting.^14^ Overfitting refers to learning the specific characteristics of training samples rather than general characteristics that can be applied to unseen data. An overfit model performs greatly on the training samples but also fails greatly on the unseen external data. 

Radiomics, which evaluates high-throughput features obtained from the images and constructs classification models based on these features, extracts features basically using 3 types of computational methods.^15^ One of these methods is shape-based features, which compute the boundary pixels and summarize the characteristics of these boundary pixel distributions related to the shape of the lesion. The second set of methods is histogram based. The histogram counts the number of pixels with each intensity level.^16^ The last method is second- and higher-order image statistics based on gray level co-occurence matrix, gray level run length matrix, gray level size zone matrix, and gray level dependence matrix.^17^ Each of these methods computes a specific property of the image and transforms the image into a set of numbers in the order of thousands. These numbers represent the underlying image and are therefore called feature representation vector. The feature vectors of all samples collectively form the feature space. For a classifier to work properly, the number of features (the dimension of feature space) should not be too high compared to the number of samples. Ideally, for each feature, there should be at least 10 samples.^18^ Therefore, the number of extracted features should be diminished before feeding them into classical machine learning classifiers. Feature selection methods are employed to discard nonrelevant features and keep the best ones.^19^ For this purpose, initially, the features that can be reproduced well by different observers are kept. These features are called robust features.^20^ Typically, correlation scores among observers are used for this selection. The second step is the elimination of redundant features. Since the features are obtained by computing different but interrelated properties of the image, they can greatly overlap or be obtained by a linear combination of the others.^20^ These will give little information together; instead, they will harm the model due to the dimensionality increase. Therefore, redundant features should be discarded, typically based on intraclass correlation coefficients. And finally, the most relevant and handful subset should remain after supervised feature selection. At the end, a robust, non-redundant, and relevant feature subset is fed into the classifiers. 

There are various studies based on classical machine learning models for stroke imaging research. A support vector machine (SVM)-based classifier was used for carotid atherosclerosis detection, and random forest-based classifier was used to detect brain edema.^21,22^ Logistic regression (LR) was used for thrombus detection in computed tomography angiography (CTA), and an artificial neural network was used for perfusion defects in computed tomography perfusion (CTP).^23,24^ Varying degrees of accuracy ranging from 85% to 97% were reached in these early studies. However, the main problem was the small sample size and lack of benchmarking opportunities against a well-curated public dataset. To mitigate this problem, various efforts are combined to publish public datasets that serve as benchmarking datasets. Furthermore, global challenges with money prizes were arranged to accelerate the solutions. Radiological Society of North America (RSNA) Intracranial Hemorrhage Detection challenge and dataset, ISLES ischemic stroke lesion segmentation challenge and dataset, and Anatomical tracings of lesions after stroke (ATLAS) challenge are some examples of the global outcomes of these efforts.^25,26,27^ In Turkey, TUSEB provided a computed tomography (CT) dataset focusing on slice-wise ischemic and hemorrhagic lesion classification task.^28^ These efforts have accelerated the production of AI applications for stroke. However, the main architecture for these applications was deep learning based rather than classical machine learning. In deep learning, a set of layers specialized in certain tasks are stacked, and relevant features are extracted by this architecture.^29,30^ The convolution, maximum pooling, and rectified linear unit (ReLU) activation functions are the backbone of deep vision models. Therefore, the models specialized on vision tasks that are heavily dependent on convolution are called convolutional neural networks (CNN).^31^ The convolution operation is matrix multiplication. A small matrix, which is usually 3 × 3 for two-dimensional images or 3 × 3 × 3 in a 3-dimensional (3D) setting and which is called a filter, traverses the image in a sliding window fashion. This filter is multiplied with the image patch under it elementwise, and the results are summed. Therefore, it transforms the underlying patch based on its values. By this way edges, corners, edges, and other high-level features could be captured, which progressively turned into low-level abstract features that represent the underlying image. The maximum pooling operation is used to find the highest peaks and keep them in order to reduce the dimension of the feature maps, and the ReLU activation function adds nonlinearity to the model to further assist complex classification tasks. Two-dimensional CNN evaluates slices, and 3D CNN evaluates the entire image volume. However, in some instances, for example, when there are not enough samples or computational resources, the image volume may require slice-by-slice encoding rather than 3D encoding. Then recurrent neural networks are utilized.^32^ Recurrent neural network (RNN) evaluates each slice like CNN and keeps that encoding to add to the next slice. Therefore, the entire image volume can be encoded slice by slice. In these architectures, a gold standard of ground truth is required. This can be stroke territory classes or stroke mechanism classes.^33^ This ground truth supervises the model while learning; therefore, this type of training is called supervised deep learning. Sometimes the ground truths cannot be at hand; therefore, supervised training cannot be applied. 

Autoencoders (AE) are self-supervised deep learning architectures.^34^ Autoencoders have 2 parts, which are the encoder and the decoder. The encoder is the same as the supervised CNN, which encodes the image into a representative vector, which is called the bottleneck layer. Since there is no explicit supervision signal, the input itself may behave as ground truth. For this purpose, a decoder part that converts the bottleneck features into the original image is added. 

For the classification tasks, the whole image volume is predicted as a certain class. However, sometimes the pathologic region may be the region of interest, and the main aim might be to segment that area from the remaining image. This type of task is called dense prediction task since each voxel in the image is predicted to be either a member of the region of interest or not. U-Net is the usual architecture for segmentation tasks.^35^ For training U-Net, which is a supervised network, a segmentation map is prepared for guiding the model. ITKSnap or 3D Slicer are free software to prepare these segmentation masks.^36,37^ U-Net architecture is like AE since it includes an encoder and a decoder. However, each down-sampled layer is reflected to the corresponding decoder level and concatenated with its output to keep the spatial information.

Deep learning methods are widely used for stroke AI research. Choi et al^38^ used RNN to predict stroke with 0.94 accuracy. Do et al^39^ used a RNN with diffusion weighted imaging (DWI) to predict MR Alberta stroke program early computed tomography score (ASPECTS) with area under curve (AUC) of 0.94. Praveen et al^40^ used AE for accurate segmentation of stroke region with 0.90 accuracy.

## Clinical and Research Applications

Various aspects of stroke clinical management can benefit from AI automatization.

Large vessel occlusion (LVO) detection, LVO location, ICH detection, CTP analysis, collateral assessment, ASPECT scoring, ischemic region segmentation, aneurysm detection, hemorrhage detection, and classification are the main targets for AI stroke research and commercialization.^41^

### Early Stroke Detection

When a patient admits the emergency room with stroke suspicion the usual first step is acquisition of non-contrast CT to rule out hemorrhagic infarct.^42^

Hemorrhage detection is an important target for stroke management. Dawud et al conducted a study with 12 635 CT images to explore binary prediction of hemorrhage of brain and reached 93% accuracy.^43^ Perreira et al^44^ utilized a CNN to classify 300 CT slices into normal, ischemic, and hemorrhagic stroke and reached 99% accuracy.

Some studies focused on ischemic stroke detection in non-contrast CT with varying degrees of performance. However, in a hyperacute setting, in less than 6 hours, CT signs are subtle for an ischemic infarct diagnosis. The authors reported over 90% accuracy with patch-wise input^45^ and in another study, the authors reported 90% accuracy with slice-wise input for ischemic stroke prediction^46^. Both studies were conducted with a small sample size, which questions their generalization ability in larger cohorts. However, in middle cerebral artery (MCA) occlusions, the thrombus inside the vessel can be seen in the hyperacute window.^47^ A hyperdense MCA sign is a good candidate for detection as a proxy for acute stroke. Lisowska et al^48^ reached 0.87 AUC to detect hyperdense MCA sign. Magnetic resonance imaging (MRI), particularly DWI, can identify ischemic lesions in the hyperacute phase.^49^ The usual routine in Turkey is rapid acquisition of DWI for confirming or ruling out stroke and then either triage to a dedicated stroke center or onsite treatment that is based on the detected pathology. The sensitivity of DWI for the diagnosis of acute stroke is 73%-92% within 3 hours of onset.^50^ Time is critical for stroke management. If the treatment is initiated within 6 hours of stroke onset, the best possible outcome can be obtained. Therefore, time to stroke onset is another important criterion while assessing stroke. In radiological evaluation, DWI-positive FLAIR-negative lesions can be regarded as in the hyperacute stage.^51^ If the lesion is well delineated in FLAIR, then odds are high that the onset of the target lesion is older than 6 hours. This is known as a DWI-FLAIR mismatch. However, more objective, and a reliable criteria are required. Shinohara et al^52^ conducted a study to detect stroke onset time and classified the patients who were within 4.5 hours of event onset with 92% precision. Abedi et al built a CNN to detect time after stroke onset and classified the patients within 4.5-hour window with a predictive AUC of 74%, which may help in stratifying patients with unwitnessed stroke episodes. Medical images have some unique properties compared to natural images. The symmetry property of the organs is among them. The symmetric organization of the brain was used by researchers to diagnose acute ischemic stroke in DWI and Apparent diffusion coefficient (ADC) maps with an AUC of 0.85.^53^

Litjens et al^54^ built a model leveraging contralateral information to extract MCA and achieved 96% AUC.

Stroke has different subtypes that require different management strategies. Therefore, the affected territory also has implications regarding the selection of treatment strategies. Subuti et al^55^ analyzed diffusion-weighted images with SVM and classified stroke territory according to total anterior, partial anterior, and lacunar infarcts with 92% accuracy. Çetinoğlu et al^56^ utilized transfer learning with EfficientNet B0 and MobileNetV2 to classify DWI images into MCA, watershed, and posterior circulation infarcts. They reported 93% overall accuracy. Lee et al^57^ designed a similar study and classified DWI images into anterior vascular zone infarcts, posterior vascular zone infarcts, and normal slices, and they reported 86% accuracy with transfer learning.

### Large Vessel Occlusion

Possible commonly known etiologies of ischemic stroke are large vessel occlusion, small vessel occlusion, and cardioembolism, according to the TOAST classification.^58^ A LVO can be reversed by using either intravenous or intraarterial thrombolysis if it can be treated earlier than 6 hours after the onset of the event.^59,60^ Although LVO accounts for 38% of acute ischemic strokes it is responsible for 60% of all stroke-related disabilities and 90% of stroke-related deaths.^61^ Computed tomography angiography is the main tool for diagnosing LVO. Amukotuwa et al^62^ utilized fast CTA to detect anterior circulation occlusions and reached 0.94 sensitivity and 0.76 specifity. Chatterjee et al^63^ used CNN to detect LVO and Shaham et al^64^ used RNN for the same task. They reached 0.82 sensitivity and 0.94 AUC, respectively, to detect LVO. Brainomix (Brainomix Ltd.), Rapid (iSchemaView), Viz (Viz.ai, California, USA), and CINA (Avicenna.ai, La Ciotat, France) are commercial products that can predict LVO.^3^ These tools also detect perfusion deficits and estimate stroke core and penumbra when they are utilized with CT perfusion images. Rapid also predicts collateral status after occlusion based on the symmetry of the vessel density of the images.^65^ McLouth et al^66^ used CINA to validate LVO^67^ and obtained 98% accuracy.

### Alberta Stroke Program Early Computed Tomography Score

While assessing the acute ischemia in CT, the exact extent of the lesion is difficult to discern due to poor margins initially. ASPECTS is developed to mitigate this difficulty and estimate severity of affection.^68^ MCA territory infarcts account for 50% of all ischemic infarcts.^69^ ASPECTS evaluates the extent of MCA infarct area by marking 10 regions of MCA feeding zone. Six of these are hemispheric regions divided into 2 compartments: cranial and caudal. The 3 hemispheric regions in either cranial or caudal division are further divided into 3 regions indicating anterior, middle, or posterior one third. The remaining 4 zones are the caudate nucleus, lentiform nucleus, insular ribbon, and internal capsule.^70^ The best score is 10, and 1 point is subtracted from the best score for each affected area to determine the final score. ASPECTS is key to define the suitability of the patient for reperfusion therapy. Currently, American Heart Association (AHA) recommends reperfusion therapy for MCA strokes with >5 ASPECTS.^71^ ASPECTS is initially defined for non-contrast CT examinations; however, it is extended to MRI as DWI-ASPECTS. There are important studies in the literature and commercial products to automatically predict the ASPECTS both from CT and DWI images. e-ASPECTS (Brainomix, Oxford, UK) and RAPID-ASPECTS (Siemens Gmbh, USA) are among them. Nagel et al^72^ designed a benchmark study utilizing RAPID-ASPECTS and e-ASPECTS against 2 radiologists. E-ASPECTS performed better than human in that study. In contrast to the findings of Nagel et al, Goebel et al^73^ found that expert consensus of radiologists performed better than e-ASPECTS which was validated by follow-up imaging. On the other hand, Guberina et al^74^ benchmarked RAPID-ASPECTS against human and found that the performance of the software is better than the human consensus. The conflicting results among these studies reflect the bias in the studies, which should be decreased in multicenter, extensive validation cohorts. The CT protocol, the age of the infarct, the experience of the radiologists, and the homogeneity of imaging protocols can affect the results. However, these AI tools exhibit considerable performance that can be adopted into the workflow with secondary supervision for increasing productivity and efficiency. In a study, authors built a CNN model to calculate the CT ASPECTS and reached 0.94 AUC for a dichotomized task that predicts ASPECTS >5.^75^ Fahed et al used RNN to predict DWI-ASPECTS and reached AUC of 0.94.^76^ Cheng et al^77^ compared AI DWI-ASPECTS and consensus of senior radiologist against junior and senior radiologists and found that software performed better than junior radiologists to correlate with the labels derived from the consensus of senior radiologists.

### Ischemic Core and Penumbra Evaluation

Perfusion is an important characteristic to assess the health of tissue, both for the brain and other organs in the body.^78^ The ischemic core is the irreversibly damaged tissue, and the penumbra is the potentially salvageable tissue in the setting of an acute ischemic infarct.^79^ One of the main purposes of revascularization therapy is to rescue the penumbra as much as possible without further compromising the status of the patients due to reperfusion injury. Therefore, the determination of core and penumbra locations and areas is important for patient management. In radiology practice, there are 2 largely utilized tools to determine the ischemia core and penumbra. One is diffusion-perfusion mismatch. Both diffusion-restricted and perfusion-deficit areas correspond to the core, and perfusion-deficit areas without diffusion restriction correspond to penumbra. The second tool is solely perfusion imaging based. In CTP imaging, the areas with a delayed time to a maximum peak more than 6 seconds correspond to the penumbra. With CTP imaging, the second tool, and with MR imaging, both tools can be used. Since CT imaging with CTA for LVO and CTP for perfusion assessment is the main working horse of stroke assessment, most of the research conducted about ischemia core and penumbra evaluation is CT based. This assessment requires segmentation of affected areas, so it is very labor-intensive. The AI segmentation tools can take place here and greatly reduce the time required for segmentation compared to manual work. Furthermore, with manual segmentation, the interobserver correlation is low, which hampers its utilization for further downstream tasks.^49^ Rapid, F-Stroke, E-Stroke, CINNA, and Vitra are some commercial software for core and penumbra evaluation.^3^ Significant differences were encountered with these software.^80^ Nevertheless, they are useful as a complementary tool. Chen et al^81^ used a 2-stage cascade CNN with DWI and reached 0.67 Dice score for stroke core segmentation, which is comparable to manual segmentation. Ho et al^82^ leveraged self-supervision with AE to locate the stroke core and penumbra on perfusion MRI and reached an AUC of 0.68. The predictive ability of deep networks for future unseen data is also a hot topic for AI research. Stroke is a dynamic process that evaluates in different directions over time. The final diseased area is one of the most important determinants of patient prognosis. Therefore, some researchers attempted to predict the final stroke volume based on the initial appearances. Nishi et al^83^ used 3D U-Net on DWI images and predicted final stroke volume with an AUC of 0.88. Yu et al^84^ approached the problem as a 2.5D task. In 2.5D approach 3 orthogonal slices are used to simulate the 3D extent of the lesion. In their work, the authors predicted the 3-7-day infarct volume by using initial MRIs and achieved a median AUC of 0.92.

### Prognosis Prediction

For the stroke patients and their families, the most important question is the expected outcome. This information is vital for the managing clinician as well, since they can advise them more confidently about the expected outcome.

The role of laboratory parameters, including biochemical and serologic biomarkers, are explored for better stroke management.^85,86^ Modified Rankin scale (mRS) is a clinical tool that assess the functional status of the patient.^87^ It is a 5-point scale with worsening a status while the score is increasing.^88^ Hoa et al^89^ built a CNN to predict mRS as a dichotomized target with cutoff score of 2 on DWI. They reached an AUC of 0.88. Ding et al^90^ developed a CNN to predict functional outcome based on infarct volume on DWI and reached an AUC of 0.97.

## Challenges

The main assumption of machine learning and deep learning studies is that the training and testing data came from the same distribution. Therefore, when a model learns the relevant features of the training set, it can be generalized to the test set. This is a strong assumption and requires a large, diverse, and comprehensive data collection strategy that covers the characteristics of the population as much as possible. However, the data regulations and privacy rules obviate collecting such data. Most of the research conducted so far has been carried out on the data from a single or a few centers with a few hundred patients at most. Therefore, all studies have bias conditioned on the distribution of the dataset at hand to some extent. This is the most important challenge that should be overcome. The solution is either the collection of large samples from different regions of the world, obtained from different scanners with different acquisition parameters, or federated learning, which implies using the data where it is acquired without taking it outside that center. The explainability and trustworthiness of the models, which consider the inherent uncertainty of medicine, are also important challenges. The models may rely on spurious correlations other than relevant features while modeling the underlying data distribution. These models may work well with that particular data; however, when data drift is encountered, they cannot produce reliable predictions. However, physicians responsibility of the patient, and their management decision, may drive the prognosis to a favorable or less favorable direction. Therefore, they are required to justify their decisions in a rational reason-outcome model. However, deep models are mainly black box models with little logic for the humans while making their predictions. Currently, saliency maps and gradient backpropagation-based solutions may help to some extent. However, better designed, more reliable, and trusted explanation tools should be developed in order to accelerate the full adoption of AI tools in radiology clinics.

## Conclusion

In conclusion, AI, including classical machine learning and deep learning, are important tools to help clinicians with stroke management. Particularly, segmentation of interest areas for further downstream tasks, which is very labor-intensive and fast triage of emergency cases in the absence of radiologists are 2 main focuses that can immediately benefit from AI models. Disease classification and decision support tools need further external validation with large cohorts and support with model explanation tools for wider adoption in radiology clinics and hospitals.
